# Prevalence of systemic diseases among patients requesting dental consultation in the public and private systems

**DOI:** 10.4317/medoral.17313

**Published:** 2011-12-06

**Authors:** Javier Fernández-Feijoo, Rafael Garea-Gorís, Marta Fernández-Varela, Inmaculada Tomás-Carmona, Marcio Diniz-Freitas, Jacobo Limeres-Posse

**Affiliations:** 1 Oral and Dental Health Unit. Fontiñas Primary Care Department. Galician Health Service (SERGAS). Spain; 2 Stomatology Department. Faculty of Medicine and Dentistry. Santiago de Compostela University. Spain And Private practice. Santiago de Compostela. Spain; 3 Private practice. Santiago de Compostela. Spain

## Abstract

Objectives: To determine the prevalence and aetiology of systemic disease among patients requesting dental treatment in public and private practice. Study Design: A retrospective analysis was performed of the medical histories of 2000 patients requesting dental treatment during the year 2009. One thousand patients came from the Fontiñas Primary Care Oral and Dental Health Unit of the Galician Health Service (SERGAS), Spain, and the other thousand from a private clinic; both clinics were situated in Santiago de Compostela, La Coruña, Spain. The data collected were the following: demographic data (age and sex), presence or absence of systemic diseases and the nosologic categories, and drug history (type and number of drugs). Results: The prevalence of systemic disease was significantly higher among patients seen in the public system (35.2% in the public system versus 28.1% in the private system; p= 0.003). The differences between the two systems were more marked when considering patients aged under 65 years, particularly with respect to rheumatic and endocrine-metabolic (diabetes) disorders. The prevalence of patients receiving polypharmacy (>4 drugs/day) was significantly higher among patients seen in the public system (5.7% in the public system versus 2.7% in the private system; p= 0.009). Conclusions: There is a high prevalence of medical disorders and of patients receiving polypharmacy among individuals requesting dental care, particularly in the public health system. Dentists must have adequate training in medical disease and must be fully integrated into primary care health teams in order to prevent or adequately resolve complications.

** Key words:** Dentistry, medical history, systemic disease, polypharmacy.

## Introduction

The advances in Medicine in recent decades, particularly with regard to early diagnosis and new therapeutic procedures, have contributed to an improvement in the quality of life of patients with chronic illness and have increased life expectancy in the general population. Spain is one of the countries of the European Community with the highest life expectancy (80.33 years in 2005) (1). This situation is also seen in oral health, contributing to the preservation of natural teeth until later in life, thus increasing the demand for dental treatment among elderly patients or those with concomitant diseases (2,3).

Some apparently healthy patients requesting dental treatment may have serious systemic disease and may be taking drugs that can influence dental treatment (4). Healthcare professionals responsible for oral and dental health of these patients must ensure that the risks of systemic complications during or as a result of dental treatment are minimised (4). Patients who come to dental clinics do not always report their past medical history, usually because they do not consider it important or do not relate it to their dental problem. An adequate medical training and the taking of a detailed medical history, which must include the patient’s past medical and drug history, and interrogation about the general state of health, are essential in order to detect patients with relevant medical conditions and to avoid the risks derived from dental treatment. With the aim of standardising this information and identifying possible medical problems, authors such as Abraham-Inpijn et al. (5) recommend the systematic administration of validated questionnaires (for example, the European Medical Risk Related History Questionnaire) to patients requesting treatment in dental clinics.

In Spain there are very few studies that have evaluated systemic disease in patients receiving dental treatment (4,6). In addition, whilst the public system offers certain basic dental care for adults and has a specialist reference departments -Stomatology and Maxillofacial Surgery- for complex cases, the private system offers all types of dental treatment though it does not usually have a specialist reference structure. The objective of this study has been to determine the prevalence and nosologic categories of systemic disease among patients requesting dental treatment, comparing the findings from the public dental service and a private dental clinic.

## Material and Methods

A retrospective review was performed of the medical histories of the 2000 patients who most recently requested dental treatment during the year 2009; one thousand histories were retrieved from a primary care oral and dental health unit of the Galician Health Service and 1000 from a private dental clinic; both clinics served an urban population in Santiago de Compostela, Galicia, Spain. The exclusion criterion was age less than 18 years; this reduced the number of evaluable histories to 1625 (671 from primary care and 954 from private practice). The variables recorded for each patient were the following: sex, age, past history of systemic diseases (the nosological categories used were cardiovascular, gastrointestinal, neurological, psychiatric, rheumatological, endocrine-metabolic, and liver and kidney disease, disability, and others) and drug consumption (patients were considered to be receiving polypharmacy if they were regularly taking more than 4 drugs).

 Statistical Analysis

The results were analysed using the SPSS statistical package version 15.0 for Windows (SPSS Inc., Chicago, Illinois, USA). Student’s t test was used to analyse age. Fisher’s exact test was used for the analysis of the variables sex, presence/absence of medical disease, nosological categories, and presence/absence of drug administration. The Mann-Whitney U test was applied to analyse differences between the private and public health systems for the variable polypharmacy. A “p” value less than 0.05 was considered statistically significant.

## Results

No statistically significant difference in the mean age was detected between patients in the public health system and private system (46.3±17.8 years and 45.7±17.5 years, respectively). In the public system, 35.2% of patients had some type of systemic disease compared to 28.1% of patients in the private clinic; this difference, which was statistically significant (p= 0.003), was due mainly to respiratory disease (3.7% in the public health system versus 1.6% in private practice, p= 0.008) and endocrine-metabolic disease (13.7% in the public system versus 6.3% in private practice, p= 0.001). Although drug consumption was similar in the two groups of patients (27% in the public system versus 23.3% in the private system), the percentage of patients receiving polypharmacy was significantly higher in the group from the public system (5.7% versus 2.7% in the private system, p= 0.009). These results are shown in ( Table 1).

Patients were then subdivided according to age (<65 years and ≥65 years). In patients under 65 years, we detected significant differences in the prevalence of systemic disease depending on the origin: 25.9% among patients in the public system and 17.6% among those from the private system (p= 0.001); this difference was due mainly to endocrine-metabolic disease (8.4% in the public system versus 3.6% in the private system, p= 0.001) and rheumatological disease (4.2% in the public system versus 2.2% in the private system, p= 0.034) ( Table 2). In patients of 65 years or older, there was no statistically significant difference in the presence or absence of systemic disease between patients in the public health service and those seen in private practice; however, on analysis of the different nosological categories, we found that patients from the public health system presented a higher prevalence of respiratory tract diseases (7.2% in the public system versus 1.2% in the private system, p= 0.011) and endocrine-metabolic diseases (36.8% in the public system versus 19% in the private system, p= 0.001) ( Table 3).

**Table 1:**
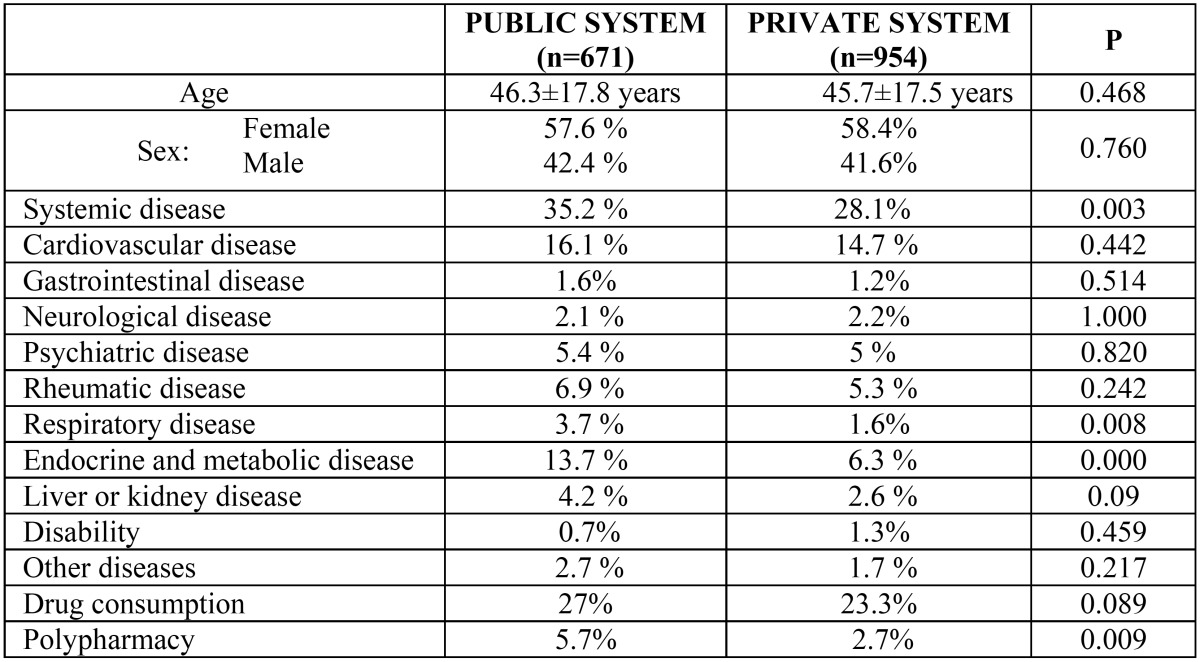
Age, sex, prevalence of systemic disease, and drug consumption among patients in the public and private health systems.

**Table 2:**
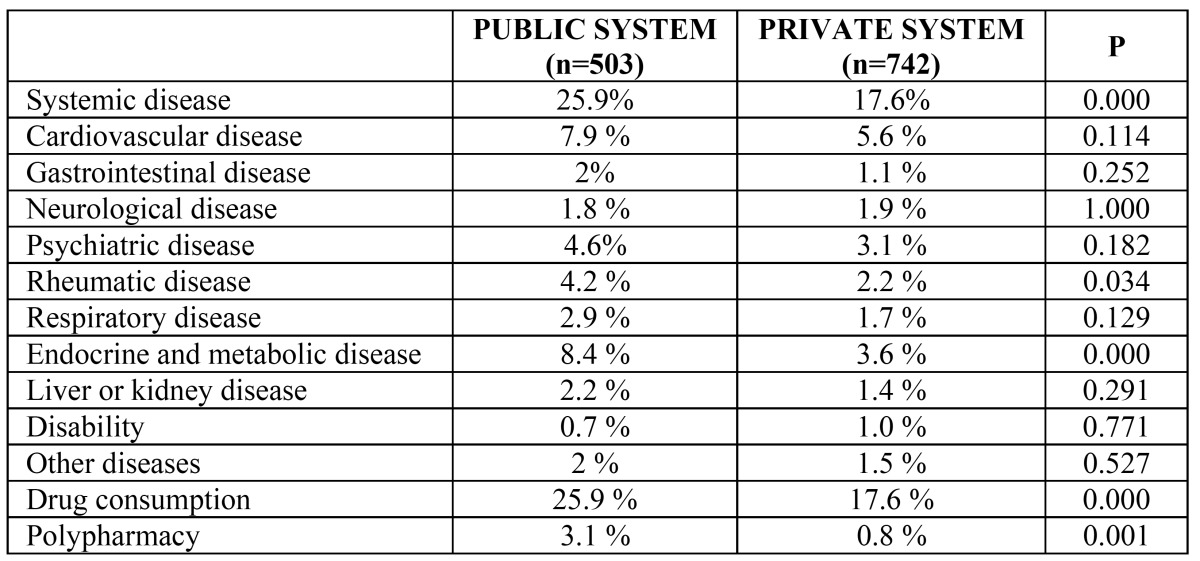
Prevalence of systemic disease and drug consumption among patients aged less than 65 years in the public and private health systems.

Among patients under 65 years of age, drug consumption was significantly higher in those from the public health service (25.9%) than in those from the private system (17.6%) (p= 0.001). The percentage of patients receiving polypharmacy was also significantly higher among those requesting dental treatment in the public health system (3.1% in the public system versus 0.8% in the private system, p= 0.001).

In the study population of 65 years or older, medication was being taken by similar proportions in the two health systems (public, 76%; private, 77.4%). In addition, there were no statistically significant differences in the percentage of patients receiving polypharmacy (16.8% in the public system versus 11.9% in the private system).

## Discussion

In the literature, the state of health of individuals requesting dental care is most commonly evaluated through the use of self-administered questionnaires that gather information on various aspects of health. However, these questionnaires have certain limitations: they require patient collaboration, they must be drawn up in a language that the patient understands, and they require confirmation of the replies by the dentist (7,8). Other authors have used a modified American Society of Anesthesiology (ASA) risk score (a method designed by the ASA in the middle of the past century to determine the risk of patients undergoing general anaesthesia) to determine the risk of dental patients treated under local anaesthesia (9,10). The objective of the present study was not to detect previously undiagnosed systemic disease but to evaluate the prevalence of known pre-existing systemic disease in a cohort of patients requesting dental treatment; we therefore used the medical histories drawn up by a single dentist.

**Table 3:**
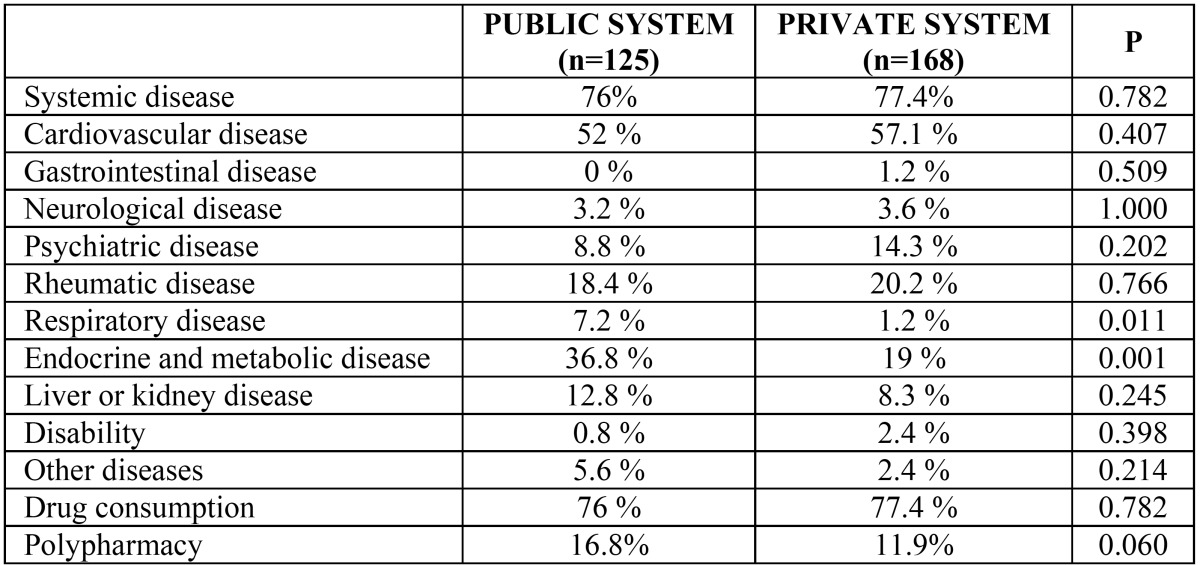
Prevalence of systemic disease and drug consumption among patients aged 65 years or older in the public and private health systems.

The prevalence of systemic disease in patients in the study group was similar to that detected by other authors, both in patients from the public system (11,12) and in those from private practice (2,4,13). The majority of diseases were of cardiovascular origin, particularly systemic hypertension (HT), as reported in previous studies (4,6, 12-15); this is compatible with the report of the National Health Survey from 2006 (16), which stated that HT was the most prevalent chronic or long-standing problem or disease diagnosed by doctors in individuals over 16 years of age in Spain. Fernández-Feijoo et al. (17), in a screening study for HT performed in a public health system dental clinic, found that 29.2% of patients requesting dental treatment had blood pressure levels suggestive of HT. This high prevalence may have been due to the fact that the authors measured the blood pressure in all patients, thus detecting patients who had not previously been diagnosed; in the present study, information was obtained from the medical histories, and we therefore only considered known hypertensive patients.

The prevalence of systemic disease among patients from the public health system was greater than among those from private practice. In the literature, the majority of studies of similar characteristics to ours were performed in university centres (4,6,12); the only study conducted on patients receiving periodontal treatment in a private clinic, a public university centre, or a hospital centre (situated in Milwaukee, Wisconsin, USA) was conducted by Nery et al. (13). Those authors also detected a higher prevalence of systemic disease among patients treated in the public centre.

In Spain, the difference in prevalence of systemic disease between public and private health systems is due mainly to a higher frequency of respiratory disease (particularly chronic obstructive pulmonary disease [COPD]) and endocrine-metabolic disease (particularly diabetes). A possible explanation for this finding is that in the primary care service where this study was performed, there was a chronic-diseases follow-up unit (diabetes, HT, COPD), in which oral health assessments formed part of the health education offered to these patients, favouring more frequent visits to the public dental clinic by patients with oral or dental health problems. Taking into account that some of these diseases have specific, early, and/or particularly aggressive oral manifestations (e.g., periodontal disease in diabetes), this could explain the higher prevalence of these disease categories in patients under 65 years requesting dental care in the public health system.

It is estimated that 75% of individuals over 55 years of age are taking some form of drug that contributes to maintaining their vital functions (18). As the number of drugs that a patient is taking rises, the risk of interactions between those drugs and medication commonly prescribed in dental practice increases (18). Our results regarding drug consumption among patients in public or private practice agree with those reported by other authors (4,19). However, the figures reported by Amado Cuesta et al. (6) and by Valderrama et al. (20) were noticeably higher; this was due to the fact that their study populations were formed only of individuals over 65 years of age and, as is widely recognised, the prevalence of medical disease and drug use increases with the age of the population (21), as was observed in our series.

Carter et al. (22), in a study in which they evaluated drug consumption among patients requesting dental treatment during the period 1984-2005, confirmed that there was a progressive increase in polypharmacy with age. In the literature, it has been shown that polypharmacy is more common in the population over 65 years of age (6,20); this was also observed in our study. The higher prevalence of patients under 65 years of age receiving polypharmacy in the public health service could be explained by the fact that the percentage of patients with systemic disease (particularly diabetes) in this age range was higher among users of the public health service. Furthermore, it may be speculated that dentists in private practice could be unwilling to treat patients receiving polypharmacy, arguing that the public health system has specialist reference departments for complex cases, or that patients themselves take this decision as they may consider that the public health system has more means to resolve any medical complication that may arise during dental treatment.

Although medical emergencies are rare in dental practice (23,24), they can occur during or as a consequence of a dental procedure and they may have a fatal outcome. The results of our study confirm that there is a significant prevalence of patients with medical disorders and who are receiving polypharmacy among individuals requesting dental treatment; this particularly affects the public health service. As a result, we believe it is essential that dentists have adequate training in medical pathology and that they are fully integrated into primary care health teams in order to prevent complications or resolve them should they develop.
